# Electrical and Optical Characterization of Sputtered Silicon Dioxide, Indium Tin Oxide, and Silicon Dioxide/Indium Tin Oxide Antireflection Coating on Single-Junction GaAs Solar Cells

**DOI:** 10.3390/ma10070700

**Published:** 2017-06-26

**Authors:** Wen-Jeng Ho, Jian-Cheng Lin, Jheng-Jie Liu, Wen-Bin Bai, Hung-Pin Shiao

**Affiliations:** 1 Department of Electro-Optical Engineering, National Taipei University of Technology, Taipei 10608, Taiwan; t104658023@ntut.edu.tw (J.-C.L.); jjliu@mail.ntut.edu.tw (J.-J.L.); mimic4442006@yahoo.com.tw (W.-B.B.); 2 Win Semiconductor Corp., Taoyuan 333, Taiwan; hpxiao@winfoundry.com

**Keywords:** antireflection, indium tin oxide (ITO), passivation, single-junction GaAs solar cells, thermally RF-sputtering

## Abstract

This study characterized the electrical and optical properties of single-junction GaAs solar cells coated with antireflective layers of silicon dioxide (SiO_2_), indium tin oxide (ITO), and a hybrid layer of SiO_2_/ITO applied using Radio frequency (RF) sputtering. The conductivity and transparency of the ITO film were characterized prior to application on GaAs cells. Reverse saturation-current and ideality factor were used to evaluate the passivation performance of the various coatings on GaAs solar cells. Optical reflectance and external quantum efficiency response were used to evaluate the antireflective performance of the coatings. Photovoltaic current-voltage measurements were used to confirm the efficiency enhancement obtained by the presence of the anti-reflective coatings. The conversion efficiency of the GaAs cells with an ITO antireflective coating (23.52%) exceeded that of cells with a SiO_2_ antireflective coating (21.92%). Due to lower series resistance and higher short-circuit current-density, the carrier collection of the GaAs cell with ITO coating exceeded that of the cell with a SiO_2_/ITO coating.

## 1. Introduction

Indium tin oxide (ITO) is among the most widely used transparent conducting oxides (TCOs) due to its high electrical conductivity and high optical transparency [1,2,3,4]. TCO films can be deposited on surfaces using various techniques, such as DC-sputtering, RF-sputtering, electron-gun evaporation, chemical vapor deposition, and spray hydrolysis. Typically, thin films of TCO are used as transparent electrodes in organic optoelectronic devices [5]. Furthermore, ITO can be applied to all types of solar cell, particularly those based on thin films [6,7,8,9]. As with all transparent conducting films, a compromise must be made between conductivity and transparency, due to the fact that any increase the thickness of the film or increase in the concentration of charge carriers to enhance conductivity decreases the transparency of the device. The electrical conductivity and optical transparency of ITO films can be improved when applied via sputtering at high temperatures in a growth environment that includes an appropriate quantity of oxygen [10,11,12,13,14]. The high electron mobility and direct bandgap of gallium arsenide (GaAs) has led to its use in high-speed RF electronics and optoelectronics [15,16]. The bandgap is close to the theoretical maximum efficiency of high-efficiency single-junction GaAs solar cells [17,18,19,20,21,22]. Dielectric films of SiO_2_, TiO_2_, or Al_2_O_3_ are typically used in the fabrication of antireflection or passivation applications involving GaAs-based solar cells [23,24,25,26]. However, few studies have simultaneously examined the passivation, antireflection, and carrier collection properties of ITO films deposited on GaAs solar cells using thermal sputtering [27,28].

In this study, we applied ITO films as a passivation/antireflection/carrier’s collection layer on single-junction GaAs solar cells with the aim of improving overall efficiency. We examined the electrical and optical properties of the ITO films as well as the dark current-voltage (I-V), optical reflectance, and external quantum efficiency of the resulting GaAs solar cells. We also confirmed the photovoltaic performances of the GaAs solar cells using photovoltaic current-voltage (I-V) measurements. We then compared the improvement in conversion efficiency following the application of an ITO AR-coating (ARC), as opposed to the application of a SiO_2_ ARC.

## 2. Experiments

**Figure 1:**
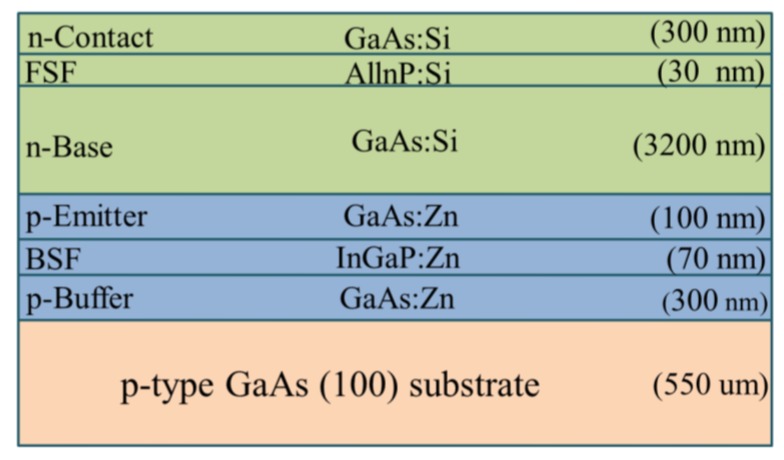
Specifications of epitaxial layers of single-junction GaAs grown via Metal-organic Chemical Vapor Deposition (MOCVD) on a p^+^-type GaAs (100) substrate.

**Figure 2:**
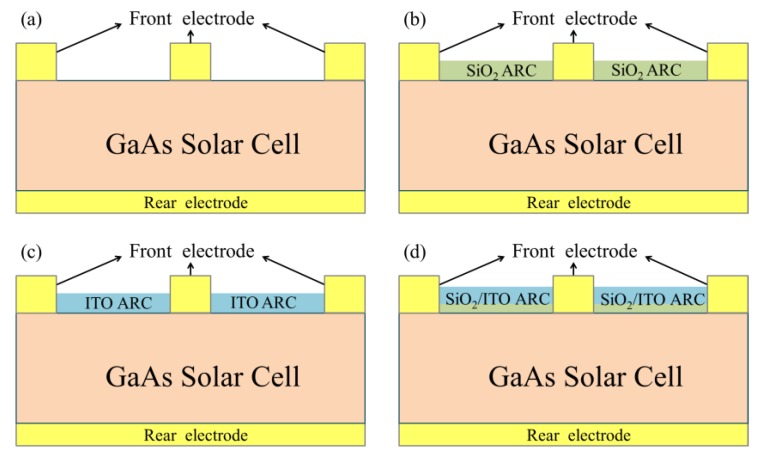
Schematic diagram showing (**a**) single-junction GaAs bare solar cell; (**b**) GaAs cell with SiO_2_ AR-coating (ARC); (**c**) GaAs cell with indium tin oxide (ITO) ARC; (**d**) GaAs cell with thin-SiO_2_/ITO ARC.

The epitaxial layers of single-junction GaAs solar cells were grown via Metal-organic Chemical Vapor Deposition (MOCVD) on a p^+^-type GaAs (100) substrate, as shown in Figure 1. Arsine and phosphine were used as group-V source gases, whereas trimethyl-gallium, trimethyl-indium, and trimethyl-aluminum were used as group-III precursors. Disilane and dimethyl-zinc were used for n- and p-type doping, respectively. Growth was conducted at a temperature of 650 °C at a chamber pressure of 20 mbar. The structure of the epitaxial layer was designed as an n-on-p cell. We first grew a p-GaAs buffer layer to a thickness of 300 nm, followed by the layers of a p-InGaP back-surface field (70 nm), a p-GaAs emitter (100 nm), a n-GaAs base (3200 nm), and an n-AlInP window as well as front-surface field (30 nm) and a n^+^-GaAs contact (300 nm). The quality of the epitaxial film was confirmed from measurements of photoluminescence, double crystal X-ray diffraction, electrochemical capacitance-voltage, and scanning electron microscopy. Fabrication of single-junction GaAs solar cells involved the deposition of a grid pattern n-ohmic contact (AuGe/Ni/Au) on the n^+^-GaAs contact layer with an AuBe/Ti/Au p-ohmic contact deposited on the rear surface of p^+^-type GaAs substrate via e-beam evaporation. The samples were then annealed at 385 °C for 5 min under ambient N_2_. Citric acid was used to selectively remove the n^+^-GaAs layer to expose the AlInP front surface field (FSF) layer, followed by mesa etching to isolate the cell (referred to as a bare solar cell). The resulting bare single-junction GaAs solar cell covered an area of 1 cm^2^, as shown in Figure 2a. To compare the antireflection and passivation characteristics of ITO film on the GaAs solar cell, we fabricated the following samples for comparison: a bare cell with a quarter wavelength thick SiO_2_ layer (SiO_2_ ARC; 94 nm), a bare cell with a quarter wavelength thick ITO layer (ITO ARC; 74 nm) and an ITO of quarter wavelength thick (74 nm) on a cell with a 20 nm SiO_2_ layer (thin-SiO_2_/ITO ARC), as respectively shown in Figure 2b–d. Experimental conditions, such as oxygen partial pressure, substrate temperature, and post deposition annealing were shown to have notable effects on the electrical and optical properties of the resulting ITO films. In this study, SiO_2_ and ITO films were deposited via RF (13.56 MHz), sputtering at a deposition rate of 0.064 nm/s, substrate temperature of 250 °C, and RF power of 40 W without post-deposition annealing. A metallic target In/Sn (90:10 wt %; 2 inch in diameter) with a purity of 99.99% was used as a source of ITO. The distance between the substrate and the target was approximately 4 cm. The average resistivity of the ITO-films was measured 3 times using a four-point probe method at room temperature. The resistivity of the ITO film was approximately 2.2 × 10^−4^ Ω·cm, and the carrier mobility was predicted to be approximately 15–20 cm^2^·V^−1^·S^−1^, which would be beneficial to photo-carrier collection.

The passivation characteristics of the SiO_2_, ITO, and thin-SiO_2_/ITO films deposited on the GaAs solar cells were characterized by measuring the dark current-voltage (I-V) curves using a semiconductor parameter analyzer (HP 4145B, Hewlett-Packard Company, Palo Alto, CA, USA) at room temperature. The antireflective properties of the SiO_2_, ITO, and thin-SiO_2_/ITO layers on the GaAs solar cells were derived from measurements of optical reflectance and external quantum efficiency response. Optical reflectance was characterized using a UV/VIS/NIR spectrophotometer (PerkinElmer LAMBDA 35, Waltham, MA, USA). External quantum efficiency (EQE) was measured over a range of wavelengths from 350 to 1100 nm, using a solar cell spectral response measurement system (EQE-RQE-R3015, Enli Technology Co., Ltd., Kaohsiung, Taiwan). The photovoltaic current density-voltage (J-V) characteristics of the proposed cells were measured using a solar simulator (XES-151S, San-Ei Electric Co., Ltd., Osaka, Japan) and source meter (Keithley 2400, Keithley Instruments, Inc., Solon, OH, USA) at 25 °C. The solar simulator was calibrated according to an NREL-certified crystalline silicon reference cell (PVM-894, PV Measurements Inc., Boulder, CO, USA) before obtaining measurements.

## 3. Results and Discussion

**Figure 3:**
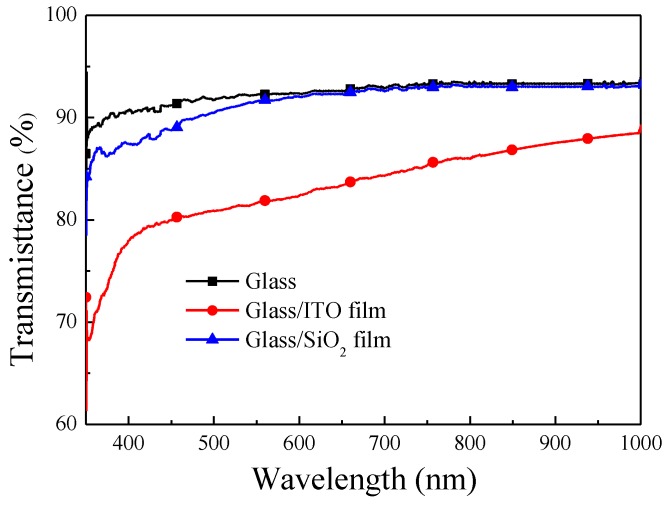
Optical transmittance of ITO film deposited by Radio frequency (RF) thermal sputtering.

**Figure 4:**
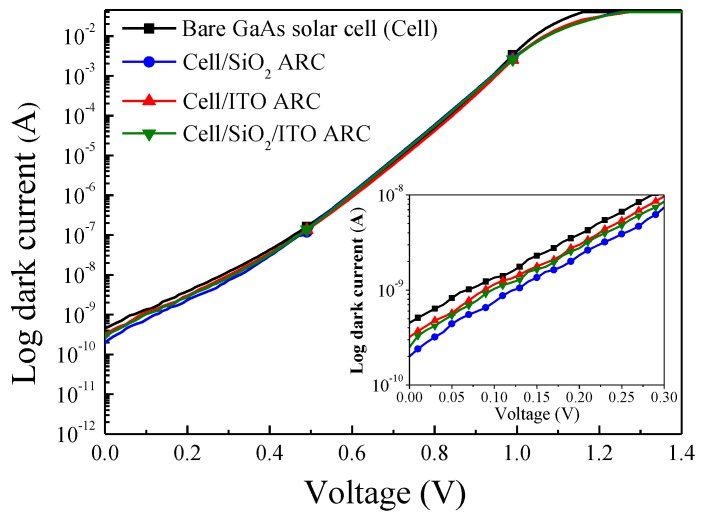
Dark I-V curves of bare GaAs solar cell, GaAs cell with SiO_2_ ARC, GaAs cell with ITO ARC, and cell with thin-SiO_2_/ITO ARC.

The refractive indexes of the RF-sputtered SiO_2_ (94 nm) and ITO (74 nm) films were 1.515 and 1.942, respectively. The average transparence of the ITO film was >83% (Figure 3). The resistivity of the ITO film was approximately 2.2 × 10^−4^ Ω·cm and the carriers mobility was predicted to be approximately 15–20 cm^2^·V^−1^·S^−1^, which would be beneficial to photo-carrier collection. Figure 4 presents the dark I-V curves of the bare GaAs solar cell, a GaAs cell with SiO_2_ ARC, a GaAs cell with ITO ARC, and a GaAs cell with thin-SiO_2_/ITO ARC. A 20 nm-thick SiO_2_ film was added to the SiO_2_/ITO configuration to identify the passivation properties of the thin SiO_2_ layer on GaAs. Reverse saturation current-density (*J*_0_) and ideality factor (*n)* were extracted from dark current-voltage curves for use in evaluating the passivation performance of the various coatings on GaAs solar cells. The calculated *n* and *J*_0_ were as follows: bare cell (2.46 and 1.27 × 10^−10^ A/cm^2^), cell with SiO_2_ layer (2.32 and 7.60 × 10^−11^ A/cm^2^), cell with ITO layer (2.42 and 9.55 × 10^−11^ A/cm^2^), and cell with thin-SiO_2_/ITO layers (2.34 and 7.68 × 10^−11^ A/cm^2^). The *J*_0_ and n results of the GaAs cell with ITO film were nearly the same as those obtained from GaAs cells with SiO_2_ or thin-SiO_2_/ITO films in which surface carrier recombination was suppressed by a SiO_2_ passivation layer. Typically, lower *n* and *J*_0_ values are indicative of superior passivation of dielectric oxide/semiconductor devices. 

**Figure 5:**
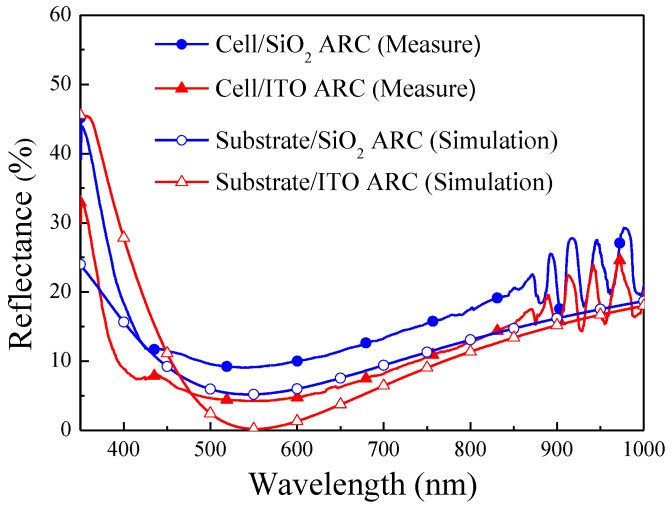
Measured optical reflectance and simulated optical reflectance of SiO_2_ ARC and ITO ARC.

**Figure 6:**
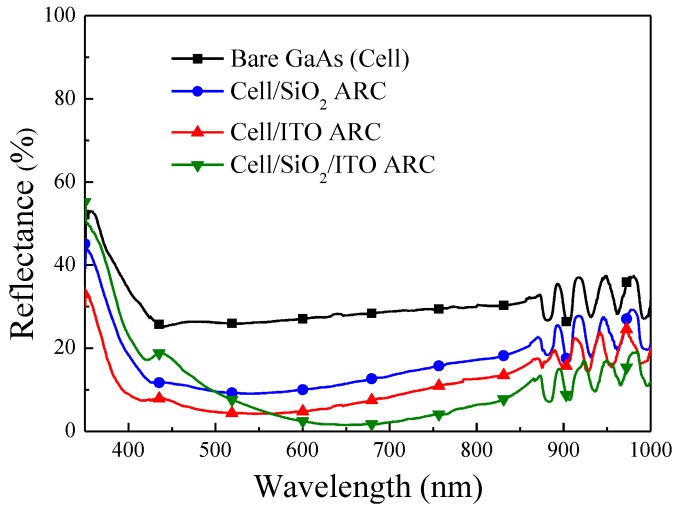
Optical reflectance of bare GaAs solar cell, GaAs cell with SiO_2_ ARC, GaAs cell with ITO ARC, and cell with thin-SiO_2_/ITO ARC.

Figure 5 presents the measured optical reflectance of GaAs cells with a SiO_2_ ARC and an ITO ARC, as well as the simulated optical reflectance of GaAs substrates with a SiO_2_ ARC and an ITO ARC. Simulations were based on data obtained from films deposited via RF sputtering. The refractive indexes of ITO (1.963), SiO_2_ (1.515) and GaAs (4.013) at a wavelength of 550 nm were used as simulation parameters. Oscillation was observed in the reflectance spectra of SiO_2_ (ITO)/GaAs solar cells over a wavelength range of 850–1000 nm, due to the resonance of light in the cavity between the epitaxial layer and substrate. The reflectance of the ITO ARC on the GaAs cell was lower than that of the SiO_2_ ARC across the entire wavelength range, thereby demonstrating the excellent anti-reflective performance of ITO films deposited using RF sputtering. The measured optical reflectance was slightly higher than the values obtained in simulations due to the presence of metal grid-electrodes on the surface of the GaAs solar cell. Figure 6 presents the optical reflectance of the bare GaAs solar cell, the cells with a SiO_2_ ARC, an ITO ARC, and a thin-SiO_2_/ITO ARC. Over the entire range of wavelengths, the reflectance of the cells with an ITO ARC and a thin-SiO_2_/ITO ARC was lower than that of the cell with a SiO_2_ ARC, thereby demonstrating the excellent anti-reflective performance of the ITO film. The average weighted reflectance (*R_W_*) values of the cell with an ITO ARC (9.29%) and the cell with a thin-SiO_2_/ITO ARC (8.90%) were lower than that of the cell with a SiO_2_ ARC (15.14%). The *R_W_* values of all evaluated cells are listed in Table 1. In the cell with a thin-SiO_2_/ITO ARC, we observed higher reflectance values at wavelengths below 570 nm, and lower reflectance values at wavelengths above 570 nm. This can be attributed to the fact that the thickness of the SiO_2_ and ITO in our thin-SiO_2_/ITO configuration was not optimized for ARC. 

**Figure 7:**
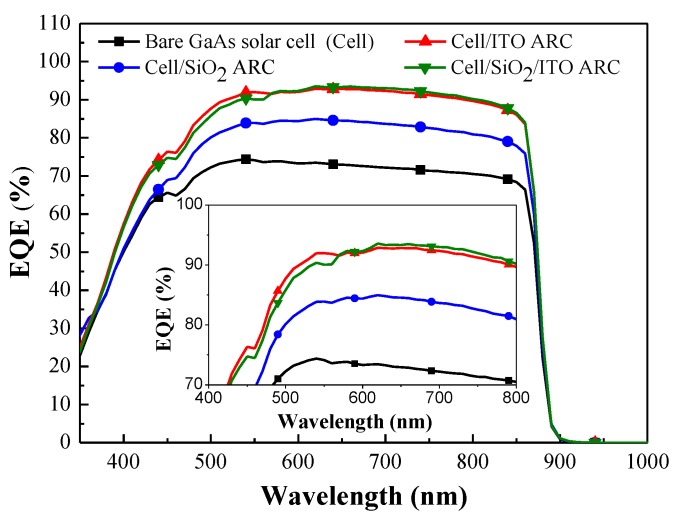
EQE response of bare GaAs solar cell and GaAs cells with ARCs of SiO_2_, ITO, or thin-SiO_2_/ITO.

As shown in Figure 7, the EQE response values of GaAs cells with an ITO ARC or a SiO_2_/ITO ARC were higher than those of the bare cell and the cell with a SiO_2_ ARC, due to lower reflectance. We calculated the average weighted EQE (*EQE_W_*) of the GaAs cells as follows: ITO ARC (72.43%), SiO_2_/ITO ARC (71.87%), SiO_2_ ARC (66.18%), and bare GaAs cell (59.03%) using Equation (1), as follows:

(1) $$EQE_{W}=\frac{\int_{350\;nm}^{900\;nm}EQE(\lambda )\varphi (\lambda )d\lambda }{\int_{350\;nm}^{900\;nm}\varphi (\lambda )d\lambda }$$

where *EQE(λ)* is the EQE response value and *φ(λ)* is the photon flux of AM 1.5G at a specific wavelength (*λ*). The *EQE_W_* values of all evaluated cells are listed in Table 1. The high *EQE_W_* of the GaAs cell with ITO ARC can be attributed to lower reflectance and a reduction in recombination loss. It should be noted that the *EQE_W_* values obtained from the GaAs cell with ITO ARC exceeded those of the cell with a SiO_2_ ARC by 6.35%. 

**Figure 8:**
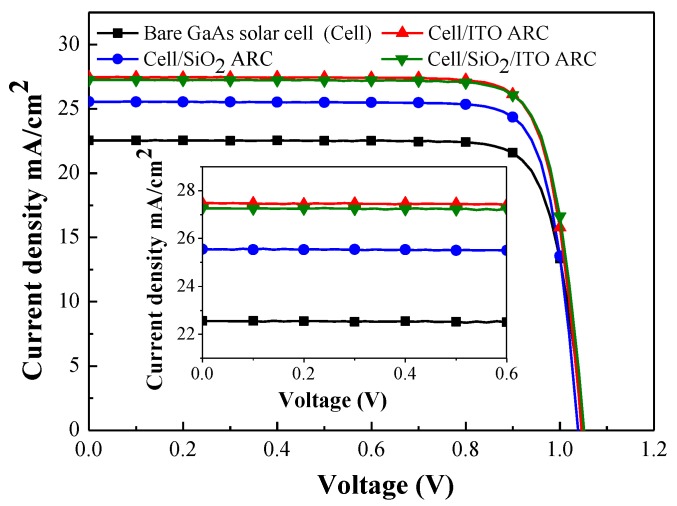
Photovoltaic current density-voltage (J-V) curves of bare GaAs solar cell and cells with ARCs of SiO_2_ ARC, ITO, and thin-SiO_2_/ITO.

Figure 8 presents the photovoltaic current density-voltage (J-V) curves of the bare GaAs solar cell, and GaAs cells with ARCs of SiO_2_, ITO, and thin-SiO_2_/ITO. Table 1 summarizes the electrical, optical, and photovoltaic performance of all cells in this study. The series resistance (*R_s_*) of the GaAs solar cell with an ITO ARC (2.55 Ω) was lower than that of the bare GaAs solar cell (3.64 Ω), the cell with a SiO_2_ ARC (3.58 Ω), and the cell with a thin-SiO_2_/ITO ARC (3.39 Ω), which indicates that an ITO layer can reduce the *R_s_* value of GaAs solar cells. The application of an ITO ARC was shown to increase the short-circuit current density (*J_sc_*) by a factor of 22.25% (from 22.47 to 27.47 mA/cm^2^) as well as the efficiency (*η*) by a factor of 22.06% (from 19.27% to 23.52%), compared to the bare solar cell. Similarly, the application of a SiO_2_ ARC increased *J_sc_* by a factor of 13.71% (from 22.47 to 25.55 mA/cm^2^) and *η* by a factor of 13.75% (from 19.27% to 21.92%), compared to the bare solar cell. This is a clear demonstration that the *J_sc_* and *η* values of the cell coated with an ITO-ARC are superior to those of the cell with a SiO_2_-ARC. Moreover, the *J_sc_* value of the cell with an ITO-ARC (27.47 mA/cm^2^) is higher than that of the cell with a thin-SiO_2_/ITO ARC (27.25 mA/cm^2^), which shows that the deposition of an ITO layer on a GaAs solar cell also promotes carriers collection. Nonetheless, the flow of photo-carriers from AlInP to ITO is small. The band offset of ITO/AlInP can be attributed to limits on carrier collection. Our laboratory is currently working to overcome this issue. In summary, these results demonstrate the strong passivation, antireflection characteristics, and carrier collection provided by the deposition of ITO films on a GaAs solar cell via thermal sputtering.

## 4. Conclusions 

In this study, we characterized the antireflection, passivation, and carrier collection characteristics of ITO films deposited on GaAs solar cells via RF sputtering. The passivation performance of the ITO film on a GaAs cell was examined in terms of reverse saturation current-density and ideality factor. Measurements of optical reflectance and EQE response demonstrate the excellent antireflective properties of the ITO ARC compared to the SiO_2_ ARC. The application of an ITO ARC was shown to increase the efficiency the GaAs solar cell by 22.05% (from 19.27% to 23.52%) and the short circuit current-density by 22.25% (from 22.47 to 27.47 mA/cm^2^). In contrast, the SiO_2_ ARC enhanced efficiency by 13.75% (from 19.27% to 21.92%), and short circuit current-density by 13.71% (from 22.47 to 22.55 mA/cm^2^). The efficiency (23.52%) and short circuit current-density (27.47 mA/cm^2^) of the GaAs cell with an ITO antireflective coating was also shown to exceed the efficiency (23.48%) and short circuit current-density (27.25 mA/cm^2^) of the GaAs cell with a hybrid antireflective layer of thin-SiO_2_/ITO, due to enhanced carrier collection.

## Figures and Tables

**Table 1:** Electrical, optical, and photovoltaic performance of all cells examined in this study.

Parameters	Bare GaAs (Cell)	Cell/SiO_2_ ARC	Cell/ITO ARC	Cell/SiO_2_/ITO ARC
*J*_0_ (A/cm^2^)	1.27 × 10^−10^	7.60 × 10^−11^	9.55 × 10^−11^	7.68 × 10^−11^
*n*	2.46	2.32	2.42	2.34
*R_W_* (%)	29.62	15.14	9.29	8.90
*EQE_w_* (%)	59.03	66.18	72.43	71.87
*J_sc_* (mA/cm^2^)	22.47	25.55	27.47	27.25
*V_oc_* (V)	1.05	1.04	1.05	1.05
*R_s_* (Ω)	3.64	3.58	2.55	3.39
*R_sh_* (Ω)	3.93 × 10^4^	5.43 × 10^5^	2.10 × 10^5^	3.05 × 10^5^
*FF* (%)	81.68	82.57	81.93	81.98
*η* (%)	19.27	21.92	23.52	23.48
